# Nitazoxanide induced myocardial injury in zebrafish embryos by activating oxidative stress response

**DOI:** 10.1111/jcmm.16922

**Published:** 2021-09-17

**Authors:** Fanghua Gong, Tianzhu Shen, Jiangnan Zhang, Xuye Wang, Guoqiang Fan, Xiaofang Che, Zhaopeng Xu, Kun Jia, Yong Huang, Xiaokun Li, Huiqiang Lu

**Affiliations:** ^1^ School of Pharmacy Wenzhou Medical University Wenzhou China; ^2^ Center for drug screening and research School of Geography and Environmental Engineering Gannan Normal University Ganzhou Jiangxi China; ^3^ Jiangxi Engineering laboratory of Zebrafish Modeling and Drug Screening for Human Diseases; Jiangxi Key Laboratory of Developmental Biology of Organs Ji’an Jiangxi China

**Keywords:** Cardiotoxicity, Nitazoxanide, Oxidative stress, RNA‐seq, Zebrafish

## Abstract

Nitazoxanide (NTZ) is a broad‐spectrum antiparasitic and antiviral drug (thiazole). However, although NTZ has been extensively used, there are no reports concerning its toxicology in vertebrates. This study used the zebrafish as a vertebrate model to evaluate the safety of NTZ and to analyse the related molecular mechanisms. The experimental results showed that zebrafish embryos exposed to NTZ had cardiac malformation and dysfunction. NTZ also significantly inhibited proliferation and promoted apoptosis in cardiomyocytes. Transcriptomic analysis used compared gene expression levels between zebrafish embryos in the NTZ treatment and the control groups identified 200 upregulated genes and 232 downregulated genes. Analysis by Kyoto encyclopaedia of genes and genomes (KEGG) and gene ontology (GO) showed that signal pathways on cardiomyocyte development were inhibited while the oxidative stress pathways were activated. Further experiments showed that NTZ increased the content of reactive oxygen species (ROS) in the hearts of zebrafish. Antioxidant gadofullerene nanoparticles (GFNPs) significantly alleviated the developmental toxicity to the heart, indicating that NTZ activated the oxidative stress response to cause embryonic cardiomyocyte injury in zebrafish. This study provides evidence that NTZ causes developmental abnormalities in the cardiovascular system of zebrafish.

## 1 INTRODUCTION

Nitazoxanide [2‐acetyloloxy‐N‐(5‐nitro‐2‐thiazolyl) benzamide] (NTZ) is a highly effective broad‐spectrum antiparasitic and antiviral drug. In the human body, NTZ is effective against a variety of intestinal parasites such as *Giardia intestinalis*, amoebae,^1^ *Cryptosporidium parvum*,^2^ trichomonas,^3^ *Blastocystis hominis* ^4^ and tapeworms.^5^ In addition, NTZ has an inhibitory effect on viral infections such as hepatitis B,^6^ hepatitis C^7^ and influenza.^8^ However, although NTZ was approved for clinical application, there is a lack of detailed reports concerning vertebrate toxicology to provide safety guidance.

The zebrafish is an emerging vertebrate model organism. This species has a high degree of homology with human genes and organs. Thus, it has become an excellent model for development biology, basic medicine and toxicology research during the last decade.^9^, ^10^, ^11^ Zebrafish embryos have unique advantages such as large yield, rapid development and optical transparency. In addition, the rapid development of imaging and transgenic technologies in recent years allows direct observation of dynamic cellular processes in the embryonic development of the zebrafish, providing detailed access to how specific genes affect development at the cellular level.^12^, ^13^, ^14^ Zebrafish are also particularly sensitive to drug exposure and are suitable for drug screening and disease modelling, facilitating the species’ key role in novel drug development.^15^

The heart is the first organ to develop in the zebrafish embryo. The zebrafish heart has a simplified structure consisting of only one atrium and one ventricle.^16^ Although anatomically different from the human heart, the zebrafish heart has analogous features including atria, ventricles, heart valves and a conduction system.^17^, ^18^, ^19^ In addition, the processes of heart development in zebrafish are very similar to those of other vertebrate models.^20^ The cardiac progenitor cells of the zebrafish are derived from the ventral marginal region of the early blastocyst and can be visualized by the expression of the cardiac regulatory myosin light chain gene (*my17*).^21^ Hence, application of the zebrafish as a research model is crucial to understand development and disease in the human heart.

There are multiple causes of heart disease, of which oxidative stress is the most important pathological mechanism.^22^, ^23^ When the production of reactive oxygen species (ROS) exceeds the antioxidant capacity of the heart, cardiomyocyte injury and dysfunction will occur. Excessive ROS adversely affects myocardial calcium (Ca^2+^), causes arrhythmia and induces apoptosis in cardiomyocytes.^24^ This study exposed zebrafish embryos to a concentration gradient of NTZ and evaluated the safety of NTZ from the perspectives of morphological changes, functional defects, cell proliferation, apoptosis and oxidative stress levels in the heart during embryonic development. Combined with transcriptome data analysis, this study provides evidence that NTZ may have potential heart developmental toxicity to the cardiovascular system.

## 2 MATERIALS AND METHODS

### 2.1 Reagents and chemicals

Nitazoxanide (99%, CAS: 55981‐09‐4) was purchased from Shanghai Yuanye Biotechnology Co., Ltd. and diluted into a stock solution of 10 mg/ml with dimethyl sulfoxide (DMSO) and stored at room temperature. The working solution was prepared by diluting the stock solution with embryo culture solution (0.3325 g/L sea salt, 4.2 mM sodium bicarbonate; pH = 7.2) before conducting the experiment. Kits for measuring enzyme activity or other biological indicators such as malondialdehyde (MDA), catalase (CAT), superoxide dismutase (SOD) and ROS were purchased from Nanjing Jiancheng Institute of Biological Engineering (Nanjing, China). RNA extraction kits, cDNA reverse transcriptase kits and Quantitative real‐time PCR (qPCR) reagents were purchased from Takara (Japan). TUNEL Apoptosis Detection Kit (Alexa Fluor 640) was purchased from Shanghai yeasen Biotechnology Co., Ltd. Gadofullerene nanoparticles (GFNPs) were kindly granted by the Institute of Chemistry, Chinese Academy of Sciences, Beijing, China. All other biochemical reagents were purchased from Sangon Biotech (Shanghai, China) and were analytically pure.

### 2.2 Zebrafish husbandry and reproduction

Tg(myl7:GFP) transgenic strain (labelled with cardiomyocytes), Tg(gata1:DsRed) transgenic strain (labelled with erythrocytes) and wild‐type AB strain were purchased from the Chinese Zebrafish Resource Center. Fish were fed with freshly hatched Artemia salina three times daily and raised under standard temperature (28 ± 1°C) and 14 h light:10 h dark photoperiod conditions according to the Institutional Animal Care and Use Committee.^25^ Promptly collect the fertilized embryos and place them in an incubator at 28°C for incubation.^26^ The 24 hpf embryos were incubated using an embryo culture containing 0.003% 1‐phenyl‐2‐thiourea (PTU) to inhibit the production of skin pigmentation, which facilitated detailed observation in subsequent experiments with microscopic imaging.

### 2.3 Drug treatment

Live embryos at 6 hpf were exposed to NTZ solutions at different final concentrations (0.5, 1.0, 2.0, 3.0, 4.0, 5.0, 6.0, 7.0 and 8.0 mg/L), and 0.1% DMSO was used as a control group. Approximately 50–60 embryos were used in each group. Embryos were cultured in a constant temperature incubator at 28°C for 90 h to 96 hpf. The fresh embryo culture medium was changed every 24 h, and embryo development indexes such as mortality, hatching and deformity rates were counted. Subsequently, we determined the final concentrations of NTZ at 0, 0.5, 1.0, 2.0 mg/L and cultured the embryos to 72 hpf for subsequent experimental treatment concentrations and times. For GFNPs rescue NTZ‐induced cardiotoxicity experiments, we used 2.0 mg/L NTZ treatment group with 500 nmol/L GFNPs for validation. A minimum of three independent replicate experiments must be performed for each experiment.

### 2.4 Morphological observation of zebrafish embryos

The exposed embryos were anaesthetized with 0.16% tricaine (Sigma, Germany) and fixed with 1% low melting point agarose in glass bottom petri dishes with the head, trunk and tail aligned on the same horizontal plane. The overall and cardiac morphology of each group of zebrafish embryos was observed and photographed using a body fluorescence microscope (Leica M205 FA stereo microscope).

### 2.5 Haematoxylin and eosin staining

Embryos from each group after exposure were collected, rinsed three times using phosphate buffer and fixed overnight in 4% paraformaldehyde (PFA), dehydrated by ethanol gradient, transparently hardened in xylene, and embedded in paraffin wax, and sectioned (7 μm) with a sectioning machine (Leica RM2235). Haematoxylin and eosin staining was performed according to the standard protocol reported previously,^27^ and the tissues were then sealed with neutral resin, covered with coverslips and dried overnight at 37°C. Images were taken and recorded using a microscope (Leica DM2500).

### 2.6 Total RNA extraction and qPCR

Total RNA was extracted from zebrafish embryos using RNAiso Plus kit. Next, approximately 1 μg of total RNA was reverse transcribed using a Prime Script^®^ RT kit to synthesize the cDNA. qRT‐PCR was performed using SYBR Green detection reagents on the ABI StepOne Plus system (Applied Biosystems). All operations were according to the manufacturer's methods. The relative mRNA levels were calculated by the 2^−ΔΔCt^ method and displayed as fold changes relative to the control group, and GAPDH was used as an endogenous control gene to normalize gene expression. The primer sequences used in this study were provided in Table S1. Each sample was tested in triplicates.

### 2.7 Antibody staining and TUNEL staining

The exposed embryos of each group were collected and fixed in 4% paraformaldehyde (PFA) overnight, and then the pericardial epicardium was peeled off under the microscope with a No. 5 forceps. Anti‐PCNA (1:100, ab71286, Abcam, UK) antibody incubation was performed as described in the previous method.^28^ Secondary antibodies were incubated using Alexa Fluor 647™ Goat anti‐mouse IgG (1: Invitrogen™). Apoptotic cells were stained using the TUNEL Apoptosis Detection Kit (Alexa Fluor 647) according to the manufacturer's instructions. Nuclei were stained for 30 min using DAPI dye. Finally, the fluorescence was imaged and photographed using a laser scanning confocal microscope (Leica TCS SP8).

### 2.8 Measurement of CAT, SOD and MDA enzyme activities and ROS

Approximately 100 foetal embryos were collected per group and the enzymatic activities of CAT, SOD and MDA in the embryos were measured using the methods described in the kit. Absorbance was measured using a SpectraMax^®^ iD3 multi‐mode enzyme marker. ROS content was measured using the fluorescent probe 2’,7'‐dichlorodihydrofluorescein diacetate (DCFH‐DA) as we described previously.^29^ The ROS content was imaged with a Zeiss body microscope (Discovery.V20) at a constant setting.

### 2.9 RNA‐seq

Approximately 40 embryos were collected for total RNA preparation, with each group including three biological replicates. The total RNA was treated with magnetic beads with OligodT enriched with mRNA with polyA tails. The obtained RNA is fragmented with interrupted buffer, reverse transcribed with random N6 primers, and then the cDNA second strand is synthesized to form double‐stranded DNA. The synthesized double‐stranded DNA ends are flattened and phosphorylated at the 5’ end, forming a sticky end with an ‘A’ protruding from the 3’ end, and then a bulbous joint with a protruding ‘T’ at the 3’ end is attached. The ligated products are amplified by PCR with specific primers. The PCR products are heat denatured to single‐stranded, and the single‐stranded DNA is then cyclized with a segmental bridge primer to obtain a single‐stranded circular DNA library. The constructed libraries were quality‐checked and then subjected to high‐throughput sequencing using the DNBSEQ platform (BGI).

### 2.10 Bioinformatics Analysis

The raw data in FASTQ format are filtered using the filtering software SOAPnuke to remove the reads with low quality, splice contamination and unknown base N content greater than 5%, and the filtered ‘Clean Reads’ are used for downstream analysis. The clean reads were aligned to the zebrafish genome sequence database using Bowtie2,^30^ genome version: GCF_000002035.6_GRCz11 (https://www.ncbi.nlm.nih.gov/), and then gene expression levels were calculated for each sample using RSEM software.^31^ Subsequently, differentially expressed genes (DEGs) had to be expressed differently than or less than 1.5‐fold using the DEseq2 method,^32^ based on the level of significance (*p*‐value <0.05). Based on the results of differential gene detection, hierarchical cluster analysis was performed for the concatenated differential genes using the R package heatmap, and Pearson correlation coefficients were calculated for the expression of all genes between each two samples. To understand the functions of DEGs in detail, gene ontology (GO) analysis was performed using the Gene Ontology database (http://geneontology.org/) to identify GO entries that were significantly enriched in candidate genes. Meanwhile, the Kyoto encyclopaedia of genes and genomes (KEGG) pathway public database was used to identify significantly enriched pathways among the candidate genes.^33^ The *p*‐values calculated during GO analysis and KEGG analysis were corrected by Bonferroni after a threshold value of *p*‐value <0.05.^34^

### 2.11 Statistical analysis

All experimental results were statistically analysed using SPSS 19.0 software, and the results were visualized using GraphPad Prism 8.0 software. For mortality, hatching rate and malformation rate statistical analyses were based on a four‐parameter logit (4 PL) regression model, including the fitting of dose‐response curves and the calculation of LC_50_.^35^ Statistical analysis of variance between control and different treatment groups was performed using one‐way analysis of variance (ANOVA), each treatment group separately from the control group using Student's t test. All values are shown as the mean ±S.D standard variance of at least three independent experiments. For all results, ^*^p < 0.05 was considered statistically significant, ^**^p < 0.01 was considered statistically significant, and ^***^p < 0.001 was considered highly statistically significant.

## 3 RESULTS

### 3.1 NTZ caused embryonic dysplasia in zebrafish

**FIGURE 1:**
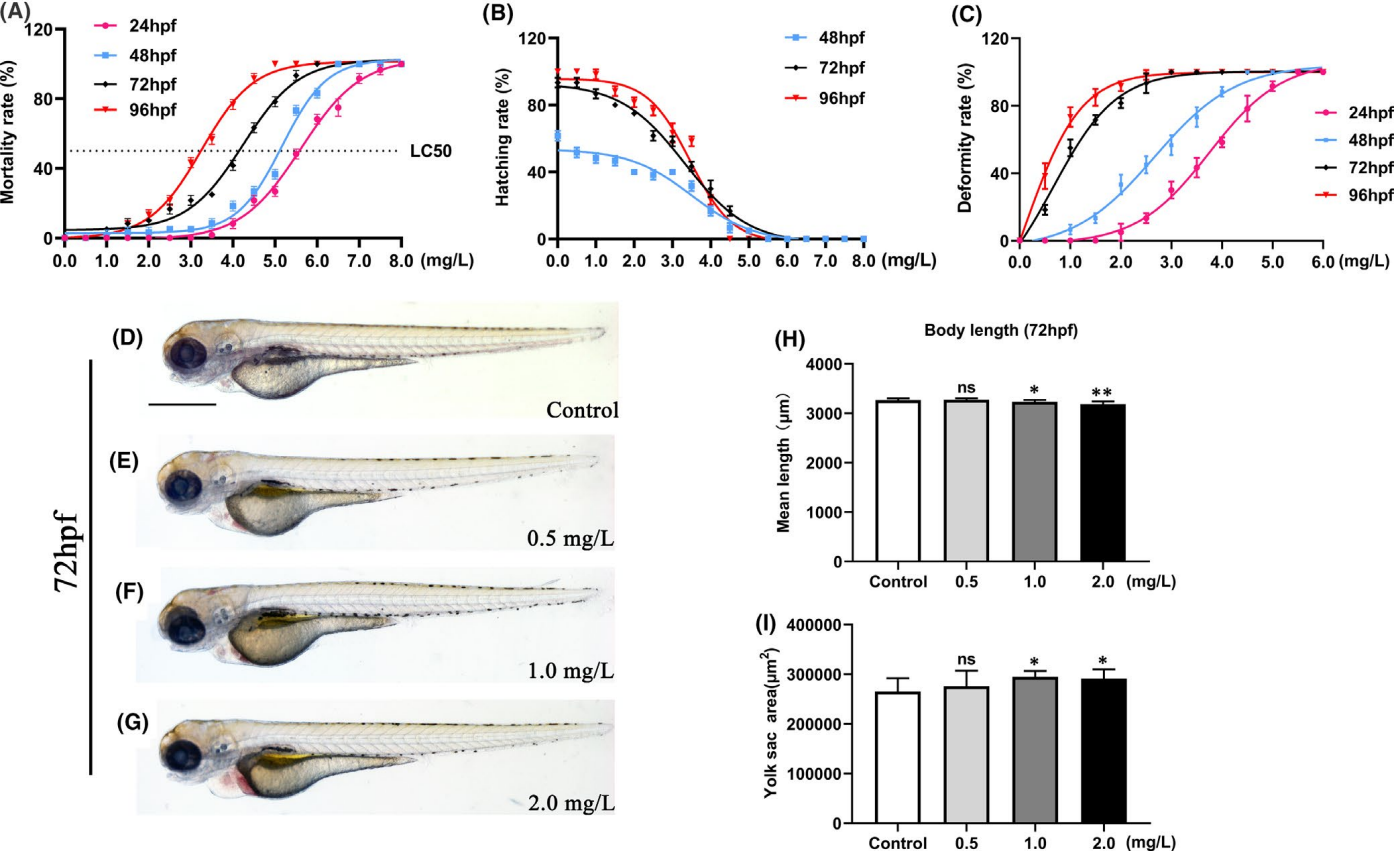
NTZ exposure induced developmental abnormalities in zebrafish embryos. Live zebrafish embryos were exposed to vehicle control (0.1% DMSO) or NTZ at various concentrations (0, 0.5, 1.0, 2.0, 3.0, 4.0, 5.0, 6.0, 7.0 and 8.0 mg/L) at 6 hpf. Statistics of (A) mortality, (B) hatching rate and (C) embryo malformation rate are shown. (D–G) Representative images of embryo morphology at 72 hpf. (H) The body length of the embryo at 72 hpf. (I) The yolk area of the embryo at 72 hpf. Note: *n* = 50 larvae per group. Scale bar: 500 μm in panels d–g. The data were presented as the mean ±standard deviation. *p < 0.05; **p < 0.01

After treating zebrafish embryos with different concentrations of NTZ, the mortality and deformity rates (Figure 1A, C) increased depending on both dosage and the timing of exposure. In addition, there was a decrease in the embryo hatching rate (Figure 1B). The embryonic half‐lethal concentration (LC_50_) values at 24 hours post‐fertilization (hpf), 48 hpf, 72 hpf and 96 hpf were 5.608, 5.166, 4.239 and 3.252 mg/L, respectively (not marked on the figure). Importantly, exposure to higher concentrations of NTZ (above 4 mg/L) directly caused the embryos to fail to develop and were lethal (Figure S1). Thus, we chose the appropriate NTZ exposure concentration (0–2.0 mg/L) and exposure time (6 hpf to 72 hpf) that would cause deformity effects without significant mortality. After treatment with different concentrations of NTZ, compared with the control group, the embryos showed different degrees of pericardial oedema and yolk sac haemorrhage (Figure 1D–G). The body length and yolk area of the embryos were also measured. The yolk sac haemorrhage of the embryos treated with moderate to high concentrations led to significant decreases in the yolk absorption rate and a larger yolk area that affected the development of the body length (Figure 1H, I).

### 3.2 NTZ‐induced embryonic heart malformations and functional defects in zebrafish

**FIGURE 2:**
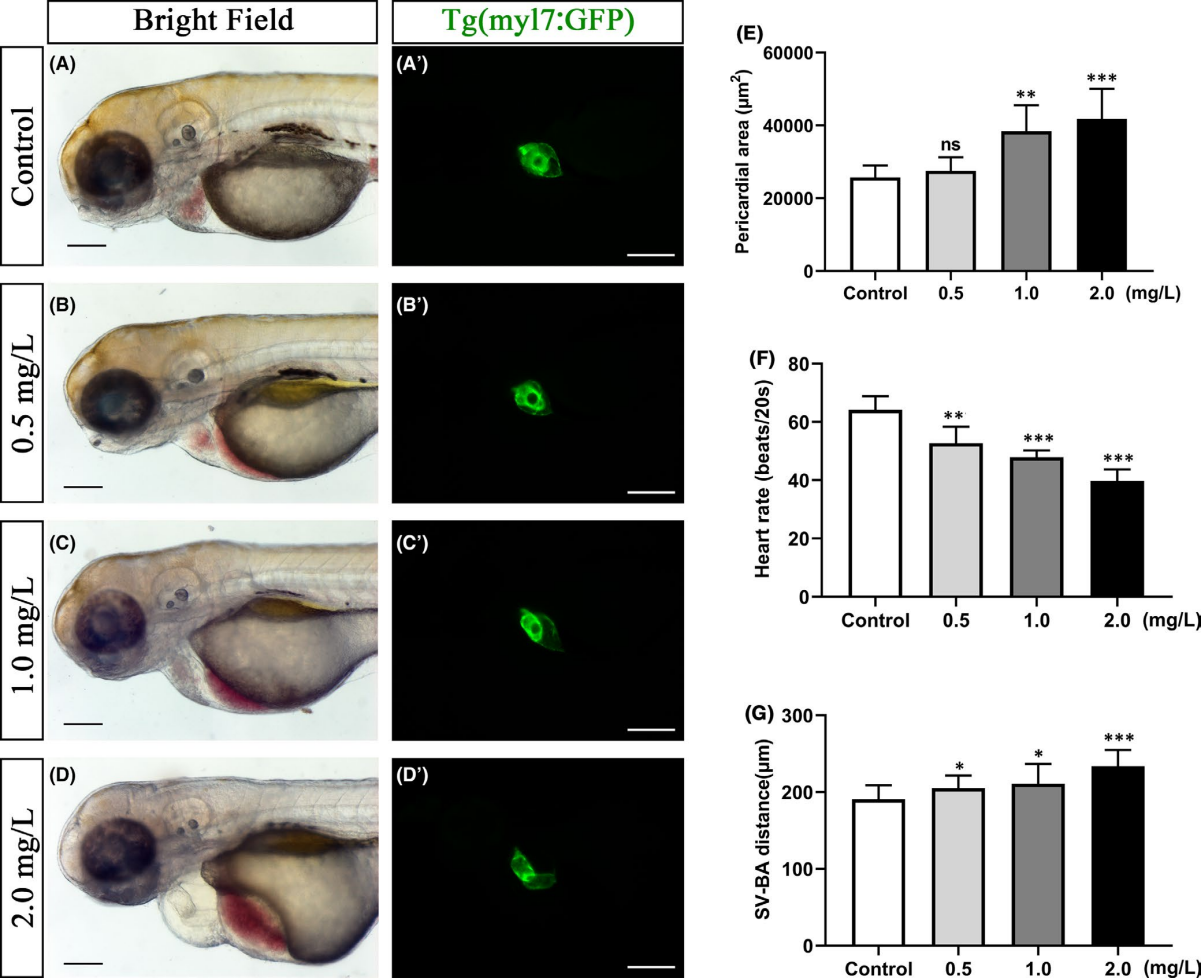
Toxic effect of NTZ exposure on embryonic heart of zebrafish. Live zebrafish embryos were exposed to vehicle control (0.1% DMSO) or NTZ at varying concentrations (0, 0.5, 1.0 and 2.0 mg/L) at 6 hpf and were observed at 72 hpf. (A–B) Enlarged high‐resolution images of the embryonic heart area in zebrafish under white light. (A'–D’) Tg (myl7: GFP) diastolic heart imaged in zebrafish embryos *in vivo*. Quantification of cardiac shape and function (E) pericardial area (μm^2^), (F) heart rate (beats/20 seconds) and (G) SV‐BA distance (μm^2^). Note: *n* = 50 larvae per group. Scale bar: 100 μm in panels a–d, a'–d’). The data were presented as the mean ±standard deviation. *p < 0.05; **p < 0.01 and ***p < 0.001

**FIGURE 3:**
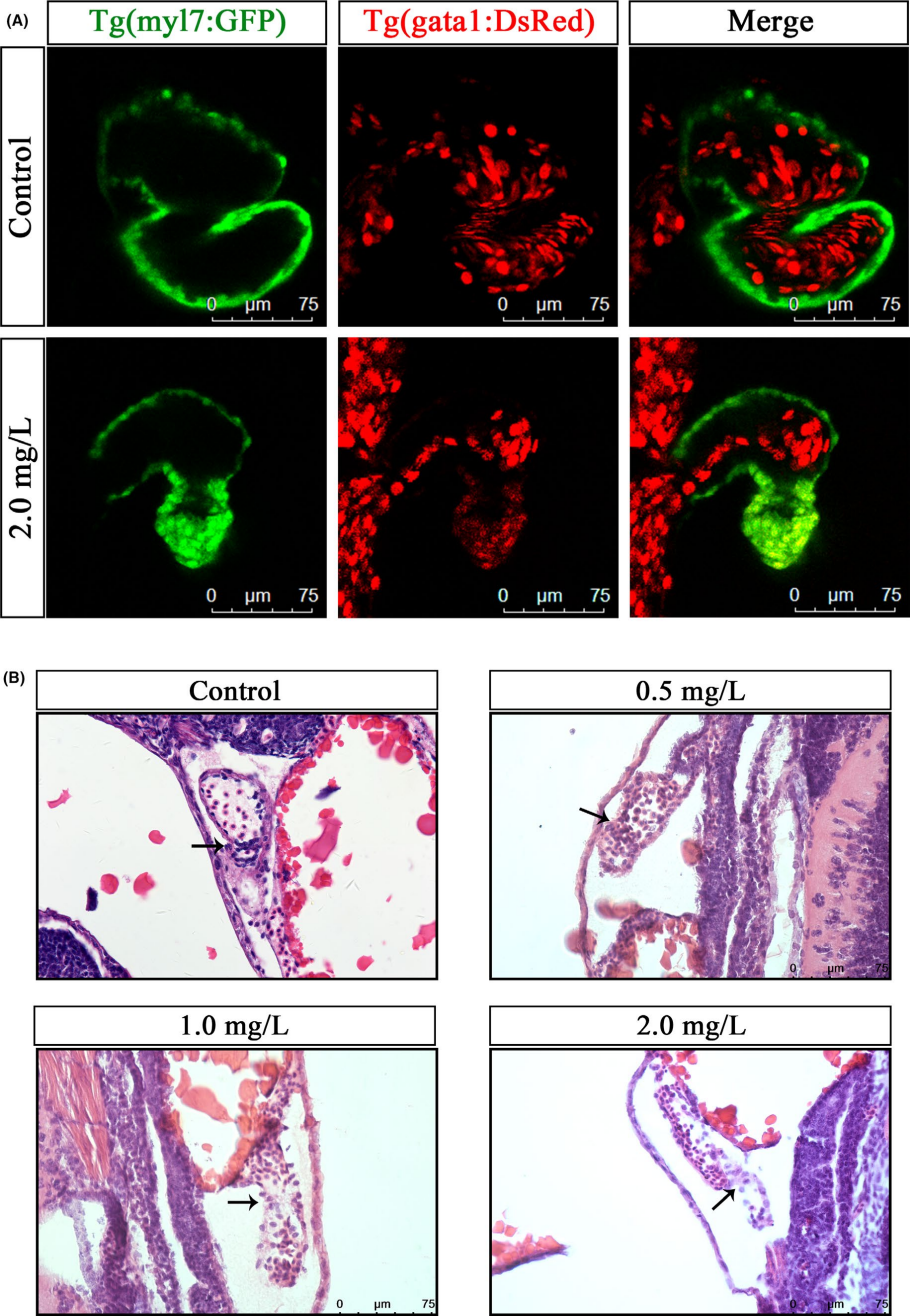
NTZ exposure reduced the pumping efficiency of the embryonic heart and severely altered the heart tissue morphology in zebrafish. (A) Heart pumping imaging of Tg (myl7: GFP) and Tg (gata1: DsRed) double transgenic zebrafish embryos. (B) Representative images of Haematoxylin and eosin staining of 72 hpf zebrafish embryos treated with different concentrations of NTZ. Note: The black arrow indicates the heart valve. Scale bar: 75 μm in panels a and b

To further examine the effects of NTZ on embryonic heart development in zebrafish, we used the Tg (myl7: GFP) transgenic strain to observe the morphological changes in cardiomyocytes. The embryos in the control group clearly had a circular heart with overlapping chambers (Figure 2A). However, the embryos treated with NTZ showed a linearized heart with little overlap between the atria and the ventricles, indicating that the heart and circulatory system had defects (Figure 2B–D; Figure S2). In addition, we determined the pericardial area, heart rate and venous sinus‐arterial bulb (SV‐BA) distance. Compared with the control group, the pericardial area of the NTZ treatment group (2.0 mg/L) was increased significantly (Figure 2E). The heart rate was significantly reduced (Figure 2F), and the SV‐BA distance was significantly increased (Figure 2G). Based on these results, we speculated that NTZ treatment may reduce the pumping efficiency of the heart of juvenile zebrafish. We used Tg (myl7: GFP) and Tg (gata1: DsRed) double transgenic fish embryos for exposure to NTZ. The results confirmed that NTZ caused a decrease in ventricular cavity volume and a defective pumping function (Figure 3A). Moreover, the results of haematoxylin and eosin staining showed that as the linearization trend between the atrium and the ventricle increased, the myocardial wall gradually became thinner, and the heart valve disappeared (Figure 3B), and the heart tissue was severely damaged. Therefore, we concluded that the morphology and function of the hearts of zebrafish embryos after exposure to NTZ were significantly altered compared with the control group.

### 3.3 NTZ affected the proliferation of cardiomyocytes and induced apoptosis

**FIGURE 4:**
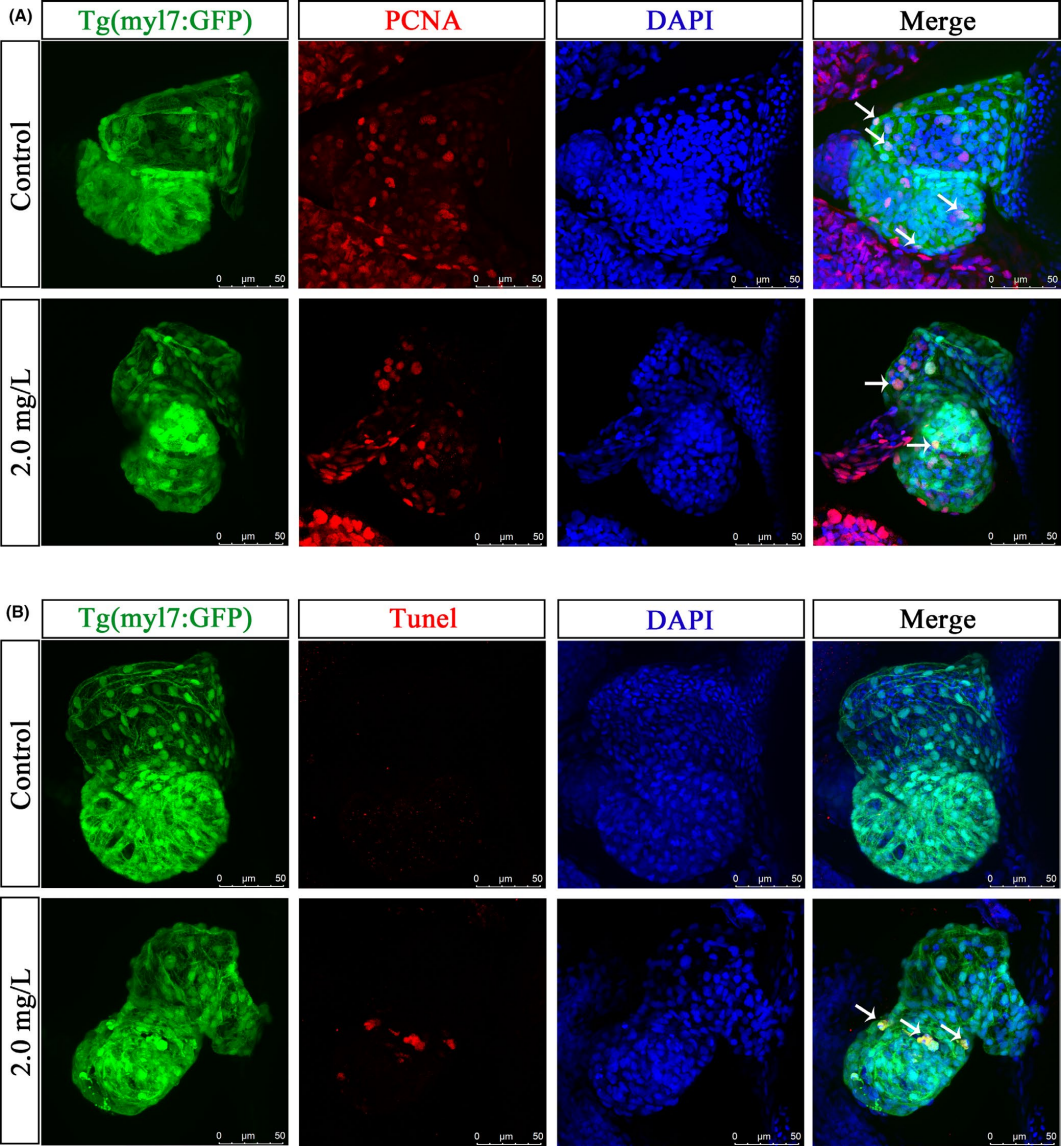
NTZ affected the proliferation of cardiomyocytes and induced apoptosis. Live Tg (myl7: GFP) zebrafish embryos were exposed to vehicle control (0.1% DMSO) or 2.0 mg/L NTZ at 6 hpf. The samples were taken at 72 hpf. (A) Representative images of fluorescence staining of PCNA showed proliferating cells. The white arrows indicate positive signals of proliferating cardiomyocytes. (B) Representative images of TUNEL staining showing apoptotic cells. The white arrow indicates a positive signal of apoptotic cardiomyocytes. Scale bar: 50 μm in panels a and b

To test whether NTZ affected the number of cardiomyocytes, we measured cell proliferation and apoptosis in the hearts of zebrafish. The results of proliferating cell nuclear antigen (PCNA) fluorescence staining showed that compared with the control group, the proliferation of embryonic cardiomyocytes treated with 2.0 mg/L NTZ was inhibited (Figure 4A). In addition, we used a terminal deoxynucleotidyl transferase dUTP nick end labelling (TUNEL) detection kit to label the apoptotic cardiomyocytes. The results showed that there were apoptotic cardiomyocytes in the heart after treatment with 2.0 mg/L NTZ, while the control group had no apoptotic cardiomyocytes (Figure 4B). These results indicated that NTZ affected normal cell proliferation and induced apoptosis in cardiomyocytes, thereby reducing the number of cardiomyocytes and affecting the normal development and function of the heart.

### 3.4 Determination and functional analysis of differentially expressed genes (DEGs) by RNA‐sequencing (RNA‐seq)

To determine the DEGs in zebrafish embryos after NTZ exposure, we performed high‐throughput RNA‐seq on the DNBSEQ platform. Before data analysis, reads with poor quality, linker contamination or high‐N content of unknown bases in the original sequencing data (Q < 20) were removed to ensure the reliability of the results. The control group produced an average of more than 44.4 million high‐quality clean reads, while the NTZ treatment group produced an average of more than 44.5 million reads. The control group had an average of 6.67 Gb of data, and the NTZ treatment group had an average of 6.71 Gb of data (Table S2). After obtaining the clean reads, the Bowtie2 tool was used to align all clean reads to the zebrafish genome sequence, with an average alignment rate of 88.81 and a total of 25,431 genes being detected.

**FIGURE 5:**
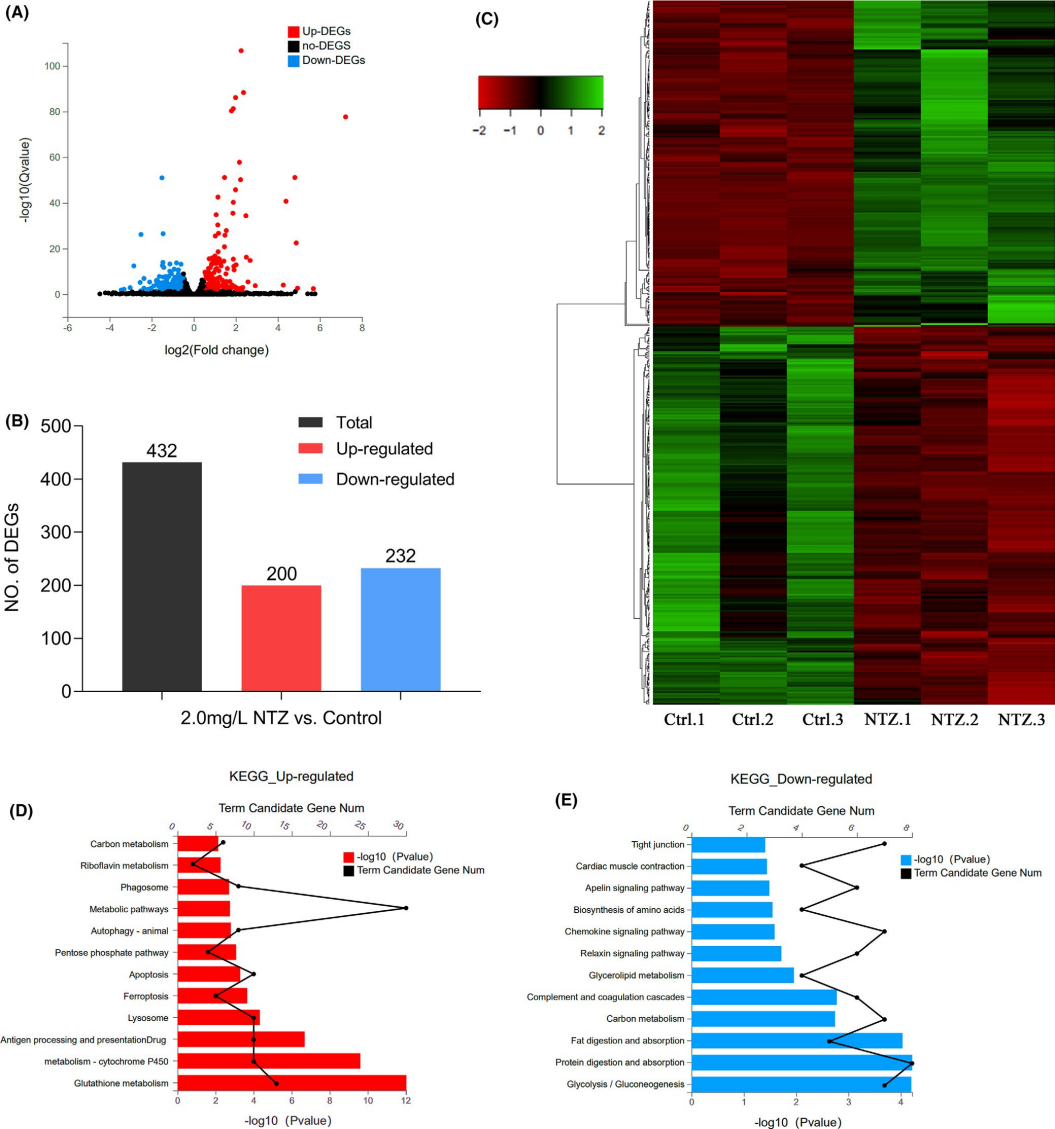
Application of RNA‐Seq to identify DEGs followed by KEGG analysis. (A) Volcano map showing DEGs identified by RNA‐sequencing. Red dots represent genes that were significantly upregulated, and blue dots represent genes that were significantly downregulated. The X‐axis represents the difference multiple value, and the Y‐axis represents the significance value. (B) Statistics of the 432 DEGs comprising 200 upregulated genes and 232 downregulated genes. (C) Hierarchical cluster analysis of DEGs in zebrafish embryos. Red represents genes that were significantly downregulated, while green represents genes that were significantly upregulated. (D) KEGG analysis of upregulated DEGs. (E) KEGG analysis of downregulated DEGs. In all analyses, the X‐axis represents the enrichment significance (*p*‐value); the Y‐axis is the KEGG pathway, and the black dots represent the number of genes enriched on a certain KEGG pathway. Each group shows the top 12 items

Subsequently, the RNA‐seq by expectation‐maximization (RSEM) software was used to calculate the gene expression levels of each sample. The control group was compared with the NTZ treatment group. There were 432 DEGs obtained, including 200 upregulated genes and 232 downregulated genes (Figure 5A, B). The threshold of screening DEGs was 1.5‐fold. A corrected *p* value <0.05 was used as the standard to distinguish the difference between the NTZ treatment group and the control group. Table S3 shows the detailed gene annotation and expression levels of DEGs. In addition, a hierarchical clustering analysis on DEGs was performed to identify two discrete expression trend clusters (Figure 5C).

### 3.5 Kyoto encyclopaedia of genes and genomes (KEGG) and gene ontology (GO) analyses of DEGs

To further understand the biological functions of DEGs, the most important biochemical metabolic pathways and signal transduction pathways involving DEGs were determined based on KEGG enrichment analysis. The 200 upregulated DEGs were classified into 27 categories with statistically significant differences (*p* < 0.05). We listed 12 categories, including ‘Glutathione metabolism’, ‘Drug metabolism ‐ cytochrome P450’ and other metabolic pathways, that were significantly enriched (Figure 5D). These results indicated that the detoxification pathway in zebrafish embryos was activated after NTZ exposure. Importantly, the ‘Apoptosis’ pathway had also been enriched. Similarly, the 232 downregulated DEGs were classified into 32 pathways. Of these, the ‘Relaxin signaling pathway’, ‘Apelin signaling pathway’, ‘Cardiac muscle contraction’ and ‘Tight junction’ pathways were enriched (Figure 5E). These pathways were involved in the developmental process and contractile function of the heart. The above results showed that the heart‐related pathways in zebrafish embryos were inhibited after NTZ exposure.

**FIGURE 6:**
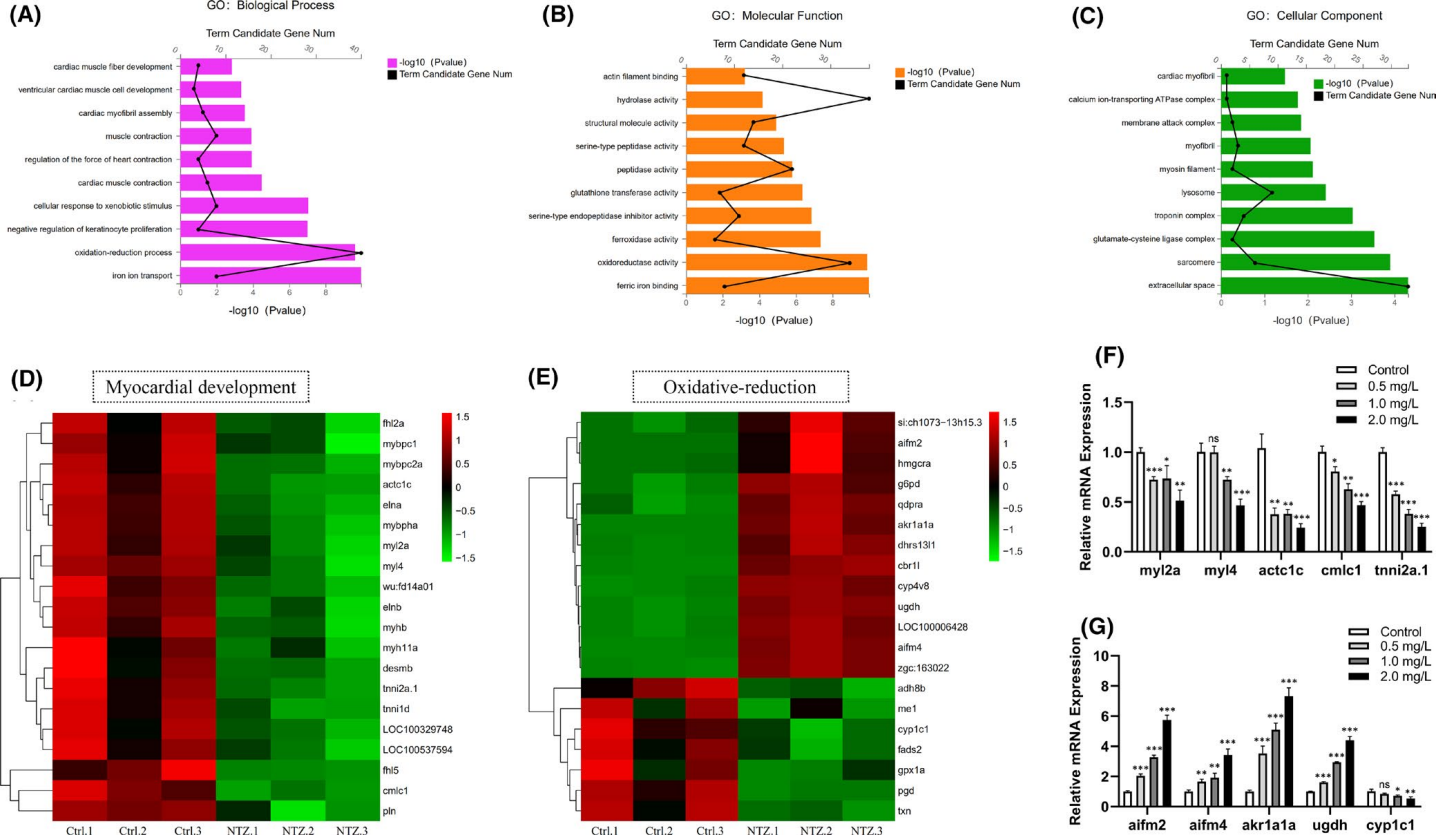
Application of RNA‐Seq for functional analysis of DEGs. (A) After NTZ exposure, the biological processes of zebrafish embryos that were significantly enriched in the GO analysis were identified. (B) After NTZ exposure, the molecular functions of zebrafish embryos significantly enhanced in the GO analysis were identified. (c) After NTZ exposure, the cell composition category was significantly enriched in the GO analysis of zebrafish embryos. In all analyses, the X‐axis represents the enrichment significance (*p*‐value). The Y‐axis lists the GO term, and the black dots represent the number of genes enriched in each GO term. Each group shows the top 10 items. (D) The heat map shows that the development of the myocardium was inhibited after NTZ exposure. (E) The heat map shows that oxidative stress has been activated after NTZ exposure. (F–G) Verification of differentially expressed genes by using qPCR. The expression levels of five randomly selected genes related to heart development and oxidative stress were confirmed. The data were presented as the mean ±standard deviation. *p < 0.05; **p < 0.01 and ***p < 0.001

Subsequently, the main biological functions performed by successfully DEGs were determined through GO function enrichment analysis. DEGs were enriched in three categories: biological process (BP); cellular component (CC); and molecular function (MF). The results showed that in the BP category, ‘oxidation‐reduction process’ had the greatest number of DEGs. DEGs were also enriched in heart‐related biological processes such as ‘cardiac muscle contraction’, ‘cardiac myofibril assembly’ and ‘ventricular cardiac muscle cell development’ (Figure 6A). Similarly, in the MF category, DEGs were enriched in many enzyme activities, especially in ‘oxidoreductase activity’, ‘hydrolase activity’ and ‘peptidase activity’ (Figure 6B). In the CC category, DEGs were mainly enriched in ‘extracellular space’ and ‘sarcomere’ (Figure 6C). These results suggested that the toxicity of NTZ to zebrafish embryos may involve activation of the oxidative stress response and may ultimately lead to cardiac dysplasia and functional defects.

### 3.6 NTZ overactivated oxidative stress and caused cardiomyocyte injury

To further explore whether NTZ cardiotoxicity was related to oxidative stress, a cluster analysis of DEGs related to myocardial development and oxidative stress was performed. Our data showed that after NTZ treatment, the expression levels of myocardial development genes were significantly downregulated (Figure 6D). In contrast, most oxidative stress genes were significantly upregulated (Figure 6E). To confirm the reliability of the RNA‐seq results, we randomly selected five DEGs from each category for qPCR analysis. The expression patterns of these genes were basically the same as in the RNA‐seq, confirming the accuracy of our results (Figure 6F, G).

**FIGURE 7:**
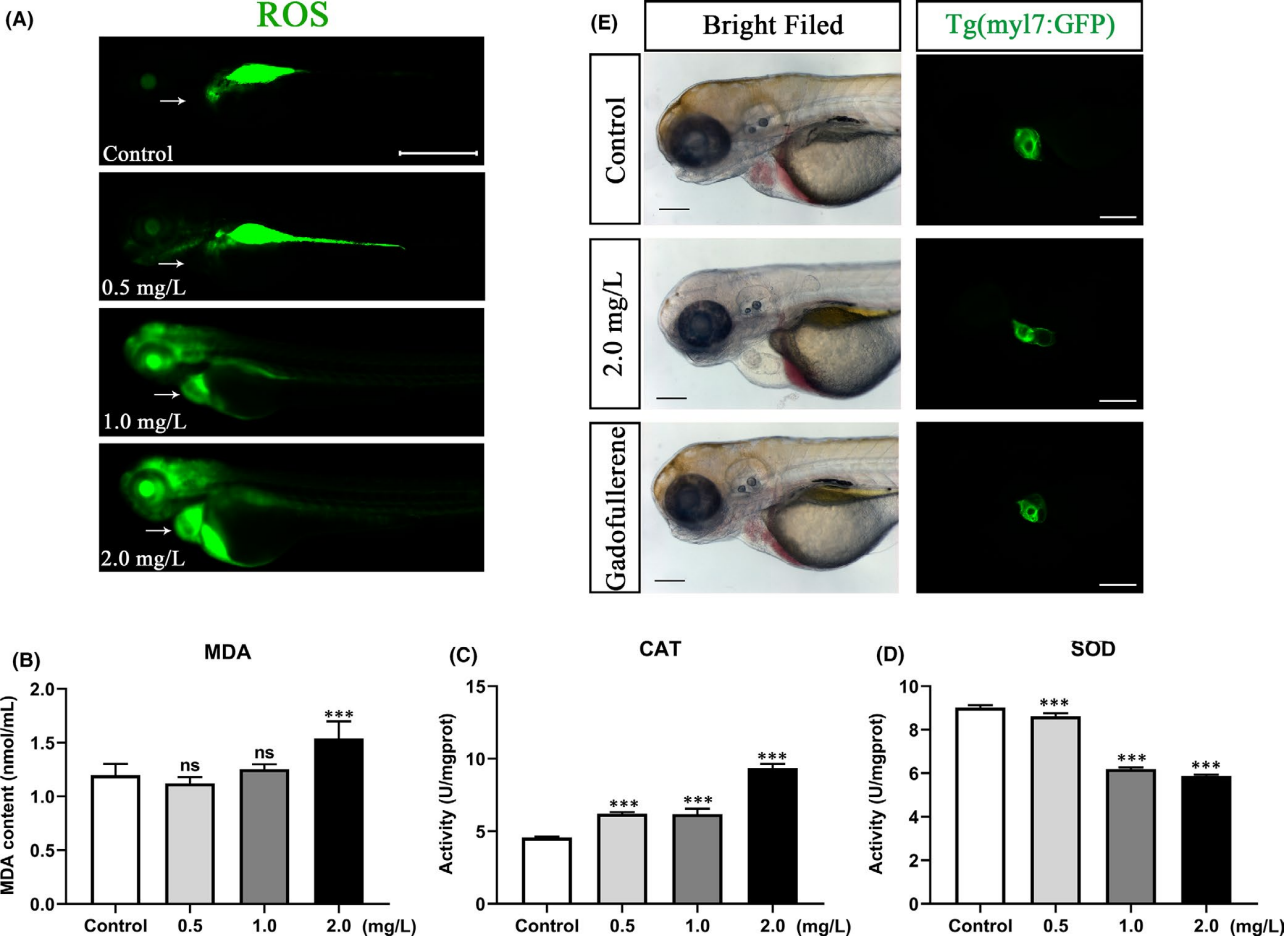
NTZ exposure caused excessive oxidative stress in zebrafish embryos. (A) ROS fluorescence distribution visualized in zebrafish embryos exposed to different concentrations of NTZ at 72 hpf. (B) MDA content, (C) CAT activity and (D) SOD activity of zebrafish embryos exposed to different concentrations of NTZ at 72 hpf. (E) Antioxidant GFNPs alleviated the cardiotoxic effects of NTZ. The data were presented as the mean ±standard deviation. ***p < 0.001

In addition, DCFH fluorescent probes were used to detect the level of oxidative stress in zebrafish embryos after NTZ exposure. The results showed that after treatment with different concentrations of NTZ, ROS levels in the hearts of zebrafish embryos were increased significantly in a dose‐dependent manner (Figure 7A). Also, NTZ treatment clearly increased the content of malondialdehyde (MDA) and the activity of catalase (CAT) in zebrafish embryos. In contrast, the activity of superoxide dismutase (SOD) in the antioxidant enzyme system was significantly inhibited (Figure 7B–D).

To confirm whether NTZ‐induced oxidative stress was the cause of cardiotoxicity in zebrafish, embryos were treated with the antioxidant GFNPs. The 2.0 mg/L NTZ treatment group with 500 nmol/L GFNPs was used for rescue verification. GFNPs ameliorated the morphological damage to the heart induced by NTZ in the zebrafish embryos. GFNPs also restored the annular heart morphology, and overlapping chambers were restored. There was no longer an excessively large pericardial area or lengthened SV‐BA distance (Figure 7E). In brief, NTZ cardiotoxicity in zebrafish embryos was alleviated by antioxidant treatment, that is excessive oxidative stress was the main pathogenic mechanism of NTZ cardiotoxicity in zebrafish embryos.

## 4 DISCUSSION

NTZ is a nitrothiazole amide compound with diverse biological activities, and it was the first drug approved by the FDA to treat *Cryptosporidium* infections.^36^ However, few publicly reported toxicology studies are available. This study selected zebrafish as an animal model to systematically evaluate the acute toxicity and molecular mechanisms of NTZ exposure in the early stages of embryonic development. Our results showed that the mortality and malformation rates of zebrafish embryos were significantly increased after exposure to NTZ. NTZ exposure also affected the hatching rate. The greatest morphological changes were in the heart, and these included pericardial oedema and heart linearization. Moreover, changes in the morphology of the heart affected normal cardiac functioning in zebrafish.

The development of the zebrafish heart is a complex and orderly process.^37^, ^38^ In short, during heart development, the myocardial progenitor cells located in the lateral mesoderm gradually converge in the midline of the abdomen and form a linear heart tube. After the proliferation of the cardiomyocytes, the chamber expands continuously accompanied by morphological changes, finally developing the subdivisions of an atrium and a ventricle.^38^, ^39^ The heart can receive blood (atrium) and pump blood (ventricle) throughout the circulatory system. In addition, the atrioventricular valve prevents blood from flowing back from the ventricle to the atrium during the entire process.^40^ Defects of the atrioventricular valve cause arrhythmia, conduction block and other effects. In this study, NTZ exposure caused severe defects in the embryonic heart development in zebrafish that included increased atrioventricular spacing and thinning of the myocardial wall, the loss of heart valves, a significant decrease in heart rate, reduced heart pumping efficiency and significant functional deterioration of the heart. The proliferation of cardiomyocytes is necessary for heart development. If the process is hindered by various factors, the number of cardiomyocytes will decrease and eventually the existing cardiomyocytes will die.^41^ Therefore, this study tested whether NTZ affected the proliferation and apoptosis of embryonic cardiomyocytes in zebrafish. The fluorescence staining with PCNA showed that NTZ hindered the normal proliferation of cardiomyocytes. In addition, the TUNEL assay confirmed that NTZ‐induced apoptosis of cardiomyocytes.

Although NTZ‐induced cardiac functional defects in zebrafish embryos, the DEGs and specific molecular mechanisms involved after exposure to NTZ were still largely unknown. High‐throughput RNA‐Seq studies gene functions at the overall level to reveal specific biological processes. Therefore, this study examined the transcription levels in the whole genome of zebrafish embryos after NTZ treatment. Bioinformatic analysis showed that there were 200 upregulated DEGs and 232 downregulated DEGs involving processes such as myocardial development, myocardial contraction and oxidative stress. In addition, we identified 20 of these genes and showed that they have often been used as biomarkers to evaluate their biological effects.

Oxidative stress is a common mechanism of cardiotoxicity. In the past few decades, nearly 10% of global drugs have been withdrawn from the market, or their clinical indications have been restricted, due to cardiovascular problems caused by oxidative stress.^42^ These drugs include doxorubicin^43^ and cisplatin.^44^ Based on our bioinformatic analyses, we speculated that NTZ‐induced cardiotoxicity in zebrafish is related to oxidative stress. Therefore, we studied the oxidative stress levels of zebrafish embryos exposed to NTZ. The results showed that NTZ exposure caused a large amount of ROS to accumulate in the heart, thereby inducing cardiomyocyte apoptosis. In addition, MDA is a commonly used peroxide indicator,^45^, ^46^ while CAT is an antioxidant enzyme that removes hydrogen peroxide and protects cells from damage.^47^ SOD is also an antioxidant that plays an important role in the balance of oxidation and antioxidation.^48^ Our results showed that MDA content was increased significantly, confirming that NTZ‐induced oxidative stress. Moreover, the activity of CAT was significantly enhanced. In contrast, SOD activity was significantly inhibited, indicating that the balance of oxidation levels in the body was disrupted. GFNPs are a kind of stable antioxidants that remove excess ROS content in the body, reduce oxidative stress levels, and inhibit apoptosis.^49^, ^50^ Consistent with this, in our study GFDPs ameliorated NTZ cardiotoxicity. This result suggested that excessive levels of oxidative stress induced by NTZ damaged cardiomyocytes.

Based on the above results, we believe that excessive oxidative stress is the key regulatory mechanism of NTZ toxicity. In addition, many metabolic processes of the zebrafish underwent significant changes after NTZ exposure. In short, this study provides new insights into the molecular mechanism of NTZ cardiotoxicity in zebrafish embryos. Future research directions should focus on unique genes in the heart or metabolic pathways and evaluate the toxic effects of NTZ from different perspectives.

## 5 CONCLUSIONS

This study has shown that exposure to NTZ affected embryonic heart development of zebrafish, significantly inhibited proliferation of cardiomyocytes, and promoted apoptosis, ultimately leading to cardiomyocyte injury. Transcriptomic analysis showed that 432 genes were differentially regulated. Metabolic pathways, redox enzymes and cardiomyocyte development were related to the NTZ toxicity. In summary, our research provides a global view of the cardiotoxicity and gene expression profile caused by NTZ exposure in zebrafish embryos. This information will help us to better understand the specific molecular mechanisms of NTZ toxicity.

## CONFLICTS OF INTEREST

The authors declare no conflict of interest.

## AUTHOR CONTRIBUTIONS

**Gong Fanghua:** Conceptualization (equal); Writing‐review & editing (equal). **Shen Tianzhu:** Writing‐original draft (equal); Writing‐review & editing (equal). **Zhang Jiangnan:** Methodology (equal). **Wang Xuye:** Software (equal). **Guoqiang Fan:** Formal analysis (equal). **Xiaofang Che:** Formal analysis (equal). **Zhaopeng Xu:** Investigation (equal). **Kun Jia:** Data curation (equal). **Yong Huang:** Data curation (equal). **Xiaokun Li:** Project administration and funding acquisition (equal). **Huiqiang Lu:** Project administration (equal).

## Supporting information

Figure S1

Figure S2

Table S1

Table S2

Table S3
