# Supplementary figures and images for: Nitazoxanide induced myocardial injury in zebrafish embryos by activating oxidative stress response
Source: J Cell Mol Med. 2021 Sep 17;25(20):9740–52. doi: 10.1111/jcmm.16922 (PMC8505840; doi:10.1111/jcmm.16922)

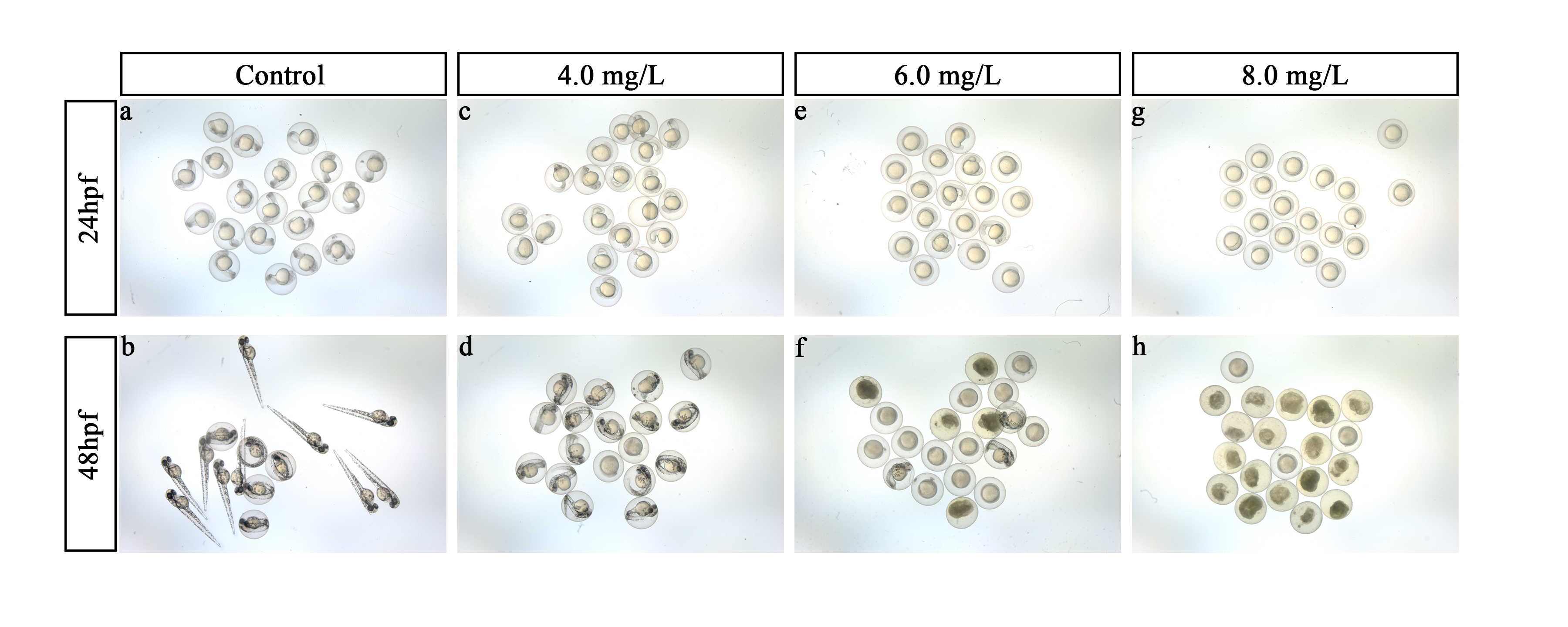

Supplement: Supplementary file 1 — Figure S1 [file JCMM-25-9740-s004.tif]

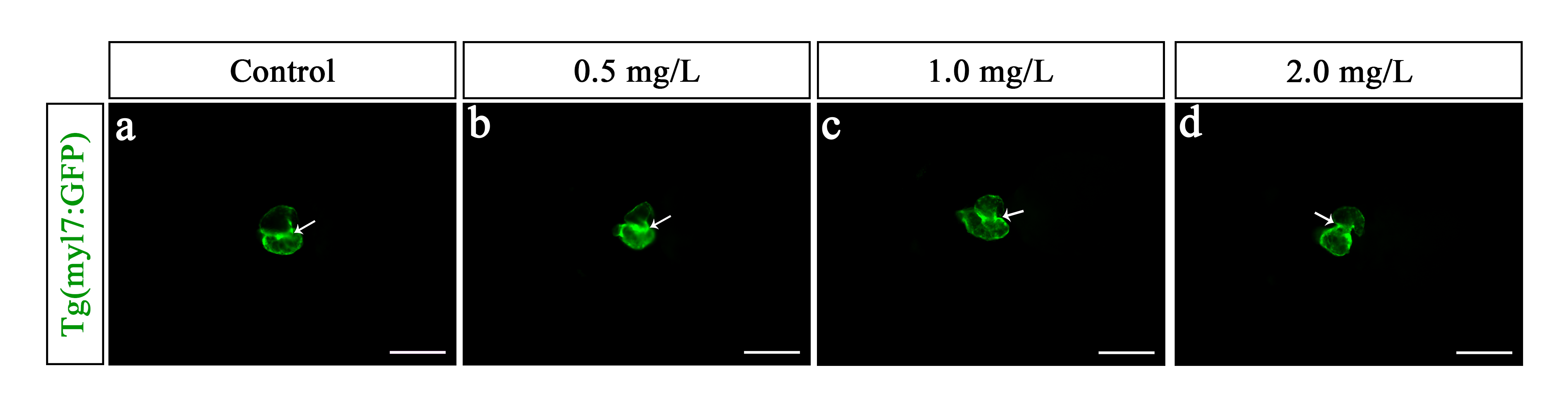

Supplement: Supplementary file 2 — Figure S2 [file JCMM-25-9740-s001.tif]
