# Supplementary material for: Nitazoxanide induced myocardial injury in zebrafish embryos by activating oxidative stress response
Source: J Cell Mol Med. 2021 Sep 17;25(20):9740–52. doi: 10.1111/jcmm.16922 (PMC8505840; doi:10.1111/jcmm.16922)
Supplement: Supplementary file 3 — Table S1 [file JCMM-25-9740-s005.docx]

| Genes | Sequence of the primers (5’-3’) | Species |
| --- | --- | --- |
| GAPDH | F：GGCACAGTCAAGGCTGAGAATG | Zebrafish |
|  | R：ATGGTGGTGAAGACGCCAGTA |  |
| Myl2a | F：GAGAGACACATTTGCAGCTCTA | Zebrafish |
|  | R：TCTGGAGGGAAAGCACTGAAC |  |
| Myl4 | F：GTTGAGTTTACAGCAGACCAGA | Zebrafish |
|  | R：CGCAAGCCCTCAACAAAGTC |  |
| Actc1c | F：AATACCCCATTGAGCATGGCA | Zebrafish |
|  | R：GCAGCGGAACCTCTCGTTA |  |
| Cmlc1 | F：TTCCAGAGACACCCAAAGAGC | Zebrafish |
|  | R：CACCCGGAGAGGATGTGCTT |  |
| Tnni2a.1 | F：ATCGAGTCGCAAGCATCATCT | Zebrafish |
|  | R：CAAACATCTTCTTCCTGCCGTC |  |
| Aifm2 | F：CAACTGGATCCTTCCCAAGCA | Zebrafish |
|  | R：CAATCGCCCACAGCATACAC |  |
| Aifm4 | F：GAGGTCACGGAGATGGTGTG | Zebrafish |
|  | R：TCACTTTGTCGTAGCAGCAC |  |
| Akr1a1a | F：TTGTGGAACACCAAGCACCA | Zebrafish |
|  | R：GATGCAAACGACGCCTCTCT |  |
| Cyp1c1 | F：GGCTGCAGTGATATCGTGGT | Zebrafish |
| Ugdh | R：CGCTTCTTACAGGGTTGGGA  F：ATAGGCGCTGGGTATGTTGG | Zebrafish |
|  | R：GCGGTCTGGCTCTTTAAGGT |  |

Supplementary Table S1. Primers were used in this study.
