# Supplementary material for: Nitazoxanide induced myocardial injury in zebrafish embryos by activating oxidative stress response
Source: J Cell Mol Med. 2021 Sep 17;25(20):9740–52. doi: 10.1111/jcmm.16922 (PMC8505840; doi:10.1111/jcmm.16922)
Supplement: Supplementary file 4 — Table S2 [file JCMM-25-9740-s002.docx]

Supplementary Table S2. Statistics of the sequencing quality of the zebrafish genome in RNA-Seq analysis.

| Sample | Total Raw Reads (M) | Total Clean Reads (M) | Total Clean Bases (Gb) | Clean Reads Q20 (%) | Clean Reads Q30 (%) | Clean Reads Ratio (%) |
| --- | --- | --- | --- | --- | --- | --- |
| Ctrl.1 | 45.57 | 44.45 | 6.67 | 97.63 | 93.73 | 97.53 |
| Ctrl.2 | 45.57 | 44.48 | 6.67 | 97.69 | 93.87 | 97.6 |
| Ctrl.3 | 45.57 | 44.43 | 6.66 | 97.34 | 93.03 | 97.48 |
| NTZ.1 | 45.57 | 44.66 | 6.7 | 97.23 | 92.73 | 98 |
| NTZ.2 | 47.33 | 44.9 | 6.74 | 97.39 | 93.2 | 94.88 |
| NTZ.3 | 45.57 | 44.52 | 6.68 | 97.32 | 93.03 | 97.69 |
